# Acute Kidney Injury After Percutaneous Left Atrial Appendage Occlusion: Incidence, Risk Factors, Clinical Impact, and Contrast-Sparing Strategies—A Systematic Review

**DOI:** 10.3390/jcm15207919

**Published:** 2026-10-14

**Authors:** Josipa Radić, Tina Bečić, Marijana Vučković, Mislav Radić, Ivana Jukić, Jonatan Vuković, Damir Fabijanić

**Affiliations:** 1 Department of Internal Medicine, Division of Nephrology, Dialysis and Arterial Hypertension, University Hospital of Split, 21000 Split, Croatia; josiparadic1973@gmail.com (J.R.); maryanchi1@gmail.com (M.V.); 2 Department of Internal Medicine, School of Medicine, University of Split, 21000 Split, Croatia; 3 Department of Cardiovascular Diseases, University Hospital of Split, 21000 Split, Croatia; tina.becic@gmail.com (T.B.); damirfabijanic62@gmail.com (D.F.); 4 Department of Internal Medicine, Division of Rheumatology, Allergology and Clinical Immunology, University Hospital of Split, 21000 Split, Croatia; 5 Department of Internal Medicine, Division of Gastroenterology, University Hospital of Split, 21000 Split, Croatia; ivjukic@gmail.com; 6 Faculty of Health Sciences, University of Split, 21000 Split, Croatia; 7 Department of Clinical Propedeutics, School of Medicine, University of Split, 21000 Split, Croatia

**Keywords:** left atrial appendage occlusion, acute kidney injury, chronic kidney disease, contrast media, zero-contrast, renal outcomes

## Abstract

**Background:** Acute kidney injury (AKI) is a clinically relevant complication following percutaneous left atrial appendage occlusion (LAAO). This systematic review evaluated its incidence, predictors, and clinical impact while separately examining the procedural feasibility and renal outcomes of contrast-sparing and zero-contrast LAAO strategies. **Methods:** PubMed, Web of Science, Scopus, and Cochrane CENTRAL were searched from database inception through 6 October 2026. Eighteen studies met the eligibility criteria. Owing to substantial clinical and methodological heterogeneity, findings were synthesized narratively, with AKI-focused evidence considered separately from contrast-sparing procedural studies. **Results:** Four studies primarily evaluated AKI following conventional LAAO. Three creatinine-based cohorts reported AKI incidences ranging from 5.3% to 13.7%, whereas one large administrative database study reported coded AKI in 5.3% of index hospitalizations; these estimates were not considered directly comparable. Pre-existing renal dysfunction was the most consistent marker of AKI susceptibility, and AKI was associated with adverse clinical outcomes. Fourteen studies evaluated contrast-sparing or zero-contrast approaches and generally demonstrated high procedural success. However, renal outcomes were inconsistently assessed; several studies did not systematically report AKI, and AKI occurred even after zero-contrast implantation. **Conclusions:** Available evidence supports the procedural feasibility of contrast-sparing and zero-contrast LAAO but does not establish that contrast avoidance prevents AKI or provides a proven nephroprotective benefit. A renal-tailored framework remains hypothesis-generating and requires prospective comparative validation using standardized renal endpoints.

## 1. Introduction

Atrial fibrillation (AF) is the most common sustained cardiac arrhythmia and a major cause of ischemic stroke and systemic thromboembolism. Oral anticoagulation (OAC) remains the cornerstone of stroke prevention; however, long-term therapy may be limited by bleeding risk, comorbidities, and contraindications to chronic anticoagulation [1]. Percutaneous left atrial appendage occlusion (LAAO) has emerged as an established non-pharmacological strategy for stroke prevention in appropriately selected patients with non-valvular AF. The PROTECT AF trial demonstrated the feasibility of percutaneous left atrial appendage (LAA) closure as an alternative to warfarin [2], while long-term data from PROTECT AF and PREVAIL supported durable protection against thromboembolic events [3]. Renal dysfunction is particularly relevant in patients undergoing LAAO. Chronic kidney disease (CKD) is associated with both increased thromboembolic and bleeding risk, complicating anticoagulation strategies in AF [4]. LAAO has been successfully performed in patients with CKD [5], including those with end-stage renal disease (ESRD) undergoing dialysis [6], although impaired renal function remains an important marker of adverse prognosis [7,8]. Recent systematic reviews and meta-analyses have primarily evaluated the overall safety and efficacy of LAAO in CKD and ESRD populations [9,10]. Beyond pre-existing CKD, AKI represents a distinct and clinically relevant complication following percutaneous LAAO. Its pathogenesis is likely multifactorial, involving baseline renal impairment, advanced age and comorbidity burden, hemodynamic and periprocedural factors, and exposure to iodinated contrast media [11]. Nombela-Franco et al. demonstrated that AKI following LAAO was not uncommon and was associated with adverse prognosis [12]. Subsequent studies further characterized its incidence, predictors, and clinical relevance [13]. In a large real-world population, Nazir et al. demonstrated a significant association between post-procedural AKI and adverse clinical outcomes, while Zhang et al. identified additional risk factors and confirmed its unfavorable prognostic implications. Collectively, these findings suggest that AKI after LAAO represents a clinically meaningful complication rather than merely a transient laboratory abnormality [14,15]. Concerns regarding renal vulnerability in patients undergoing LAAO have stimulated the development of contrast-sparing and contrast-free procedural strategies. Echocardiography-guided LAAO without iodinated contrast has demonstrated procedural feasibility compared with conventional contrast-guided implantation [16]. Subsequent studies expanded this approach using hybrid echocardiography–fluoroscopy guidance [17], specifically evaluated zero-contrast LAAO in patients with CKD [18], and demonstrated contrast-free implantation with alternative occluder platforms, including the LAMBRE device [19]. More recent real-world and comparative experience has further supported the technical feasibility and procedural safety of zero-contrast LAAO [20,21]. These developments demonstrate that iodinated contrast can be minimized or avoided in selected patients; however, procedural feasibility should not be interpreted as evidence that contrast avoidance prevents AKI. Despite these advances, the available evidence remains fragmented. Previous systematic reviews have predominantly addressed the overall safety and efficacy of LAAO in CKD or ESRD populations [9,10], whereas evidence specifically addressing post-LAAO AKI varies substantially in definitions, ascertainment methods, populations, risk factors, and reported outcomes. In parallel, the expanding literature on contrast-sparing and zero-contrast LAAO has primarily focused on procedural feasibility and safety, with renal outcomes inconsistently assessed. This systematic review therefore aimed to synthesize the evidence on the incidence, predictors, and prognostic implications of AKI following percutaneous LAAO and, separately, to evaluate the procedural feasibility and safety of contrast-sparing and zero-contrast strategies. Within the latter body of evidence, renal outcomes were examined where explicitly reported, with particular attention to whether the available data supported conclusions regarding AKI prevention.

## 2. Materials and Methods

### 2.1. Study Design and Reporting Standards

This systematic review was conducted and reported in accordance with the Preferred Reporting Items for Systematic Reviews and Meta-Analyses (PRISMA) 2020 statement [22]. The review protocol was registered in the International Prospective Register of Systematic Reviews (PROSPERO; registration number: CRD420261500407). The review addressed two complementary bodies of evidence: studies evaluating the incidence, predictors, and clinical consequences of AKI following percutaneous LAAO, and studies evaluating the procedural feasibility and safety of contrast-sparing or zero-contrast LAAO strategies. For the revised synthesis, these bodies of evidence were explicitly distinguished, and procedural feasibility and safety were assessed separately from reported renal outcomes and evidence regarding AKI prevention.

### 2.2. Search Strategy

A comprehensive updated literature search was performed in PubMed, Web of Science, Scopus, and the Cochrane Central Register of Controlled Trials (CENTRAL) from database inception through 6 October 2026. Embase was not searched because institutional access was unavailable at the time of the updated search. The search strategy combined terms related to percutaneous left atrial appendage occlusion/closure with terms describing acute kidney injury, renal dysfunction, chronic kidney disease, and contrast-sparing or zero-contrast procedural approaches. The search was deliberately broadened in the revised review to capture studies that might have been missed by the more restrictive renal terminology used in the original search, particularly studies evaluating contrast-free procedural feasibility in patients with impaired renal function.

The core search concept combined terms for LAAO/LAAC and relevant devices (including WATCHMAN, WATCHMAN FLX, Amulet, Amplatzer Cardiac Plug, LAmbre, WaveCrest, and Ultraseal) with renal and contrast-related terms, including acute kidney injury, acute renal injury or failure, renal or kidney impairment/dysfunction, chronic kidney disease, renal insufficiency, contrast-associated or contrast-induced acute kidney injury/nephropathy, nephrotoxicity, zero-contrast, contrast-free, low-contrast, and contrast-minimization strategies. Database-specific syntax, field tags, and controlled vocabulary were adapted as appropriate for each database. The complete database-specific search strategies are provided in Supplementary Table S1.

No language restrictions were applied. No study-design filter was used. The updated search retrieved 849 records: 247 from PubMed, 312 from Web of Science, 269 from Scopus, and 21 from CENTRAL. Reference lists and citation networks of eligible studies and relevant reviews were additionally examined to identify potentially eligible studies not captured by the electronic database search; this process identified one additional study that fulfilled the inclusion criteria.

Duplicate records were identified by matching publication titles and available bibliographic information across databases. Titles were normalized for minor formatting differences, including punctuation, spacing, hyphenation, and trademark symbols, and potentially matching records with minor title variations were manually verified before removal. Following deduplication, 367 duplicate records were removed, leaving 482 unique records for title and abstract screening.

### 2.3. Eligibility Criteria and Review Questions

Two complementary but distinct review questions were prespecified for the revised synthesis.

Review question 1: AKI after LAAO (PECO framework). The population (P) comprised adults undergoing percutaneous LAAO/LAAC. The exposure (E) was postprocedural AKI or patient- and procedure-related factors potentially associated with AKI, including baseline renal dysfunction and procedural characteristics. The comparator (C), when available, consisted of patients without AKI or patients across different renal-risk or exposure categories. The outcomes (O) included AKI incidence, predictors and associated factors, need for renal replacement therapy, mortality, major adverse cardiovascular events, procedural complications, and hospital readmission. Studies were eligible for this question if they reported an identifiable AKI or clinically relevant postprocedural renal outcome after LAAO.

Review question 2: Contrast-sparing or zero-contrast LAAO (PICO framework). The population (P) comprised adults undergoing percutaneous LAAO/LAAC. The intervention (I) was a procedural strategy designed to reduce or eliminate the use of iodinated contrast, including zero-contrast, contrast-free, low-contrast, or other contrast-sparing approaches. The comparator (C), when available, was conventional contrast-guided LAAO; uncontrolled single-arm studies were also eligible because the feasibility literature largely consisted of observational series. The outcomes (O) included procedural feasibility and success, periprocedural safety, device-related outcomes, contrast use, and, when reported, AKI or other postprocedural renal outcomes. Studies without a separately reported renal endpoint were eligible for this question if they provided original data on the feasibility or safety of a prespecified contrast-sparing strategy; however, such studies were not considered evidence of renal protection.

Eligible study designs included prospective or retrospective cohort studies, registry-based analyses, comparative non-randomized studies, and case series providing sufficient original cohort-level data. Studies evaluating commercially available percutaneous LAAO devices were eligible, and no language restrictions were applied.

Studies were excluded if they: (1) focused exclusively on CKD or end-stage renal disease without reporting AKI-specific/postprocedural renal outcomes and without evaluating a contrast-sparing procedural strategy; (2) evaluated interventions other than percutaneous LAAO/LAAC; (3) were single case reports; (4) were reviews, editorials, guidelines, commentaries, or other publications without original patient data; or (5) represented duplicate, superseded, or substantially overlapping reports of an already included study population. When multiple reports described overlapping populations, the most complete and informative report was retained.

### 2.4. Study Selection

The updated database search identified 849 records, including 247 from PubMed, 312 from Web of Science, 269 from Scopus, and 21 from Cochrane CENTRAL. After removal of 367 duplicate records, 482 unique records underwent title and abstract screening. Of these, 461 records were excluded because they did not meet the predefined eligibility criteria.

Twenty-one reports were sought for full-text retrieval. One potentially eligible report could not be retrieved in full text, leaving 20 reports for full-text eligibility assessment. Following full-text evaluation, three reports were excluded because they represented superseded or overlapping conference reports corresponding to subsequently published or more complete study populations. Thus, 17 studies identified through database searching fulfilled the eligibility criteria.

**Figure 1:**
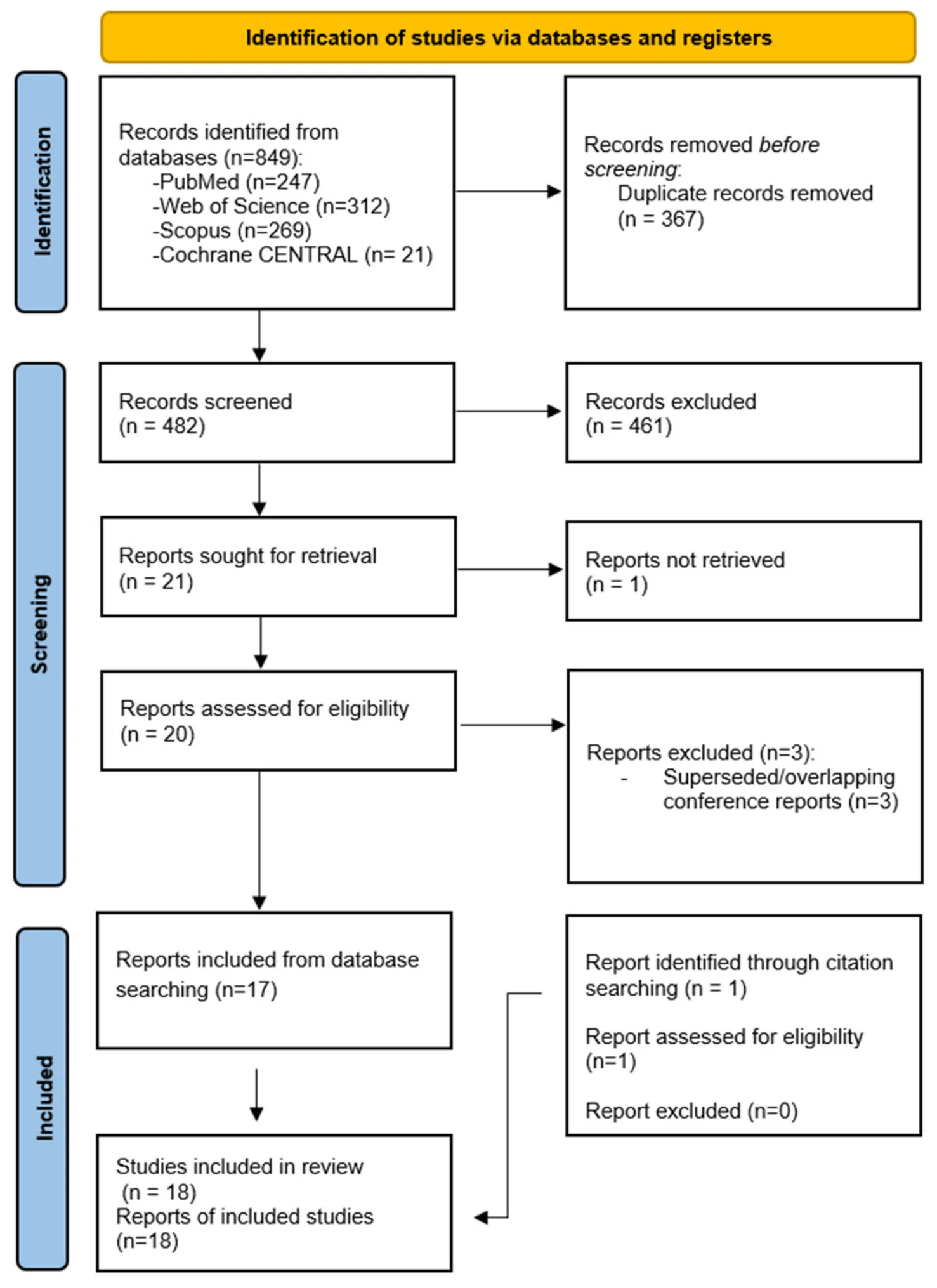
PRISMA 2020 flow diagram illustrating the study identification, screening, eligibility assessment, and final inclusion process. AKI, acute kidney injury; CKD, chronic kidney disease.

In addition, one eligible study was identified through citation searching and was included after full-text assessment. Overall, 18 studies were included in the final systematic review. Study selection was performed independently by two investigators (JR and TB), and disagreements were resolved through discussion and consensus. The updated study selection process is summarized in the PRISMA 2020 flow diagram (Figure 1).

### 2.5. Data Extraction and Outcomes

Data extraction was performed independently by two investigators (JR and TB) using a standardized data extraction framework. Discrepancies were resolved by consensus. The following information was extracted whenever available: first author and publication year, study design, sample size, patient population, baseline renal function, definition of AKI, type of LAAO device and procedural strategy, use and volume of iodinated contrast media, incidence of AKI, predictors of AKI, changes in serum creatinine or estimated glomerular filtration rate (eGFR), procedural success, periprocedural complications, mortality, major adverse cardiovascular events (MACE), and duration of follow-up. The outcomes of interest were organized into four prespecified domains reflecting the objectives of the review: (1) incidence of AKI following LAAO; (2) patient- and procedure-related risk factors for AKI; (3) clinical and prognostic impact of AKI; and (4) renal and clinical outcomes associated with contrast-sparing or zero-contrast LAAO strategies. Where individual studies used different definitions of AKI, the definition reported by the original investigators was retained and extracted rather than retrospectively applying a uniform definition.

### 2.6. Risk-of-Bias Assessment

The methodological quality and risk of bias of the included studies were independently assessed by two investigators (JR and TB) using design-specific Joanna Briggs Institute (JBI) critical appraisal tools.

The JBI Critical Appraisal Checklist for Cohort Studies was applied to cohort and comparative observational studies, whereas the JBI Critical Appraisal Checklist for Case Series was used for uncontrolled single-arm studies. Relevant domains included participant selection, ascertainment of exposure and outcomes, identification and management of potential confounding factors, completeness of follow-up, and appropriateness of statistical analyses.

Each domain was assessed as Yes, No, Unclear, or Not applicable, as appropriate. Disagreements between reviewers were resolved by consensus. Risk-of-bias assessments were considered at the domain level and incorporated into the interpretation of the overall evidence rather than relying solely on an aggregate numerical quality score.

Item-level judgments for each JBI critical appraisal question are presented separately for cohort/comparative studies and uncontrolled case series in Supplementary Tables S2A and S2B, respectively. No aggregate numerical quality score was calculated.

### 2.7. Data Synthesis

Given the substantial clinical and methodological heterogeneity across the included studies—including differences in study design, patient populations, baseline renal function, AKI definitions and ascertainment windows, LAAO devices, contrast exposure, procedural imaging strategies, and reported outcomes—a structured narrative synthesis was performed. The evidence was organized according to two distinct but complementary review questions. The first body of evidence comprised studies evaluating the incidence, predictors, and prognostic consequences of AKI following LAAO. The second comprised studies evaluating contrast-sparing or zero-contrast LAAO strategies, with procedural feasibility and safety considered separately from explicitly reported renal outcomes.

Within the AKI-focused evidence, studies were compared according to the definition and timing of AKI ascertainment, baseline renal function, reported contrast exposure, adjusted or unadjusted predictors, and subsequent clinical outcomes. Creatinine-based AKI definitions were distinguished from AKI identified solely through administrative diagnostic coding. Within the contrast-sparing/zero-contrast evidence, studies were further categorized according to whether they included a contemporaneous contrast-guided comparator or represented uncontrolled single-arm series. Studies that did not systematically assess AKI contributed only to conclusions regarding procedural feasibility and safety and were not interpreted as evidence of renal protection. Similarly, changes in eGFR, reports of acute kidney disease, and absence of reported renal events were retained using the terminology of the original investigators and were not treated as equivalent to standardized AKI ascertainment.

No numerical weighting scheme was applied. Instead, greater interpretive weight was assigned to evidence from studies with a contemporaneous comparator, standardized renal outcome definitions, clearly specified assessment windows, appropriate adjustment for potential confounders, larger sample sizes, and lower risk of bias. Findings from small, uncontrolled case series or studies without systematic renal outcome ascertainment were interpreted more cautiously and primarily as evidence of procedural feasibility. Where available, numerical estimates of AKI incidence, measures of association for potential predictors, renal function parameters, mortality, major adverse cardiovascular events, procedural success, and other relevant clinical outcomes were extracted and reported as presented in the original studies.

Because of substantial heterogeneity in AKI definitions, ascertainment periods, patient selection, baseline renal risk, procedural strategies, and outcome reporting, quantitative pooling was considered inappropriate and no summary effect estimate was calculated. To facilitate visual comparison without implying statistical pooling, study-specific AKI incidences with 95% confidence intervals, calculated using the Wilson score method, were presented in a forest-style plot for studies providing extractable event counts and denominators. No pooled estimate was calculated because of substantial clinical and methodological heterogeneity.

Because a formal GRADE assessment was not prespecified, certainty was not assigned using formal GRADE categories. Instead, the strength of evidence supporting each major conclusion was interpreted qualitatively according to study design, risk of bias, consistency of findings, directness and standardization of renal outcome ascertainment, sample size, and the availability of a contemporaneous comparator. Particular caution was applied to conclusions regarding renal protection with contrast-sparing or zero-contrast strategies because many of these studies were uncontrolled and did not systematically ascertain AKI.

## 3. Results

### 3.1. Study Selection

The updated database search identified 849 records across PubMed, Web of Science, Scopus, and Cochrane CENTRAL. After removal of 367 duplicate records, 482 unique records underwent title and abstract screening, of which 461 were excluded. Twenty-one reports were sought for full-text retrieval; one report could not be retrieved, leaving 20 reports for eligibility assessment. Three reports were excluded because they represented superseded or overlapping conference reports corresponding to subsequently published or more complete study populations. Seventeen studies identified through database searching fulfilled the eligibility criteria. One additional eligible study was identified through citation searching, resulting in a total of 18 studies included in the systematic review. The study-selection process is summarized in the PRISMA 2020 flow diagram (Figure 1).

### 3.2. Characteristics of the Included Studies

The 18 included studies were published between 2018 and 2026 and comprised a heterogeneous body of observational evidence, including multicenter and single-center cohorts, registry-based analyses, propensity-matched and nonrandomized comparative studies, and uncontrolled case series. Sample sizes ranged from 10 patients in the contrast-free WATCHMAN FLX series by Okada et al. to a weighted national estimate of 20,703 patients in the administrative database analysis by Nazir et al. [14,23].

The evidence comprised two complementary but distinct bodies of literature. Four studies primarily evaluated the incidence, predictors, and prognostic implications of AKI following conventional LAAO [12,13,14,15]. Fourteen studies primarily evaluated contrast-sparing or zero-contrast procedural strategies, including comparative and uncontrolled feasibility studies. These studies were interpreted separately according to whether they provided procedural feasibility and safety data, systematically assessed renal outcomes, or both. Studies without systematic AKI ascertainment were not considered evidence of renal protection.

Devices and procedural approaches varied across studies and included Amplatzer/Amulet, WATCHMAN/WATCHMAN FLX, and LAMBRE platforms, with different combinations of transesophageal or intracardiac echocardiography, fluoroscopy, and electroanatomic guidance. The principal characteristics of the included studies are summarized in Table 1.

### 3.3. Incidence of AKI Following LAAO

Three clinical cohorts assessed postprocedural AKI using standardized serum creatinine-based criteria. Nombela-Franco et al. applied AKIN criteria within 48 h and reported AKI in 32 of 355 patients (9.0%) [12]. Sedaghat et al. likewise used creatinine-based AKIN criteria, comparing baseline creatinine with the peak value within 48 h after LAAO, and reported AKI in 13 of 95 patients (13.7%) [13]. Zhang et al. applied KDIGO serum creatinine criteria, using the peak serum creatinine value within 7 days after LAAO, and reported AKI in 27 of 512 patients (5.3%) [15]. Thus, among studies using creatinine-based ascertainment, reported AKI incidence ranged from 5.3% to 13.7%.

Administrative evidence was considered separately. Nazir et al. identified AKI using ICD-10-CM diagnostic codes during the index hospitalization in 1097 of 20,703 patients (5.3%) [14]. Serum creatinine data were unavailable and the timing of AKI relative to LAAO could not be determined. This estimate therefore represents the frequency of coded AKI during the index hospitalization rather than a confirmed incidence of new postprocedural AKI.

The observed incidence range should be interpreted descriptively and not as a pooled estimate. Differences in AKI definitions, ascertainment windows, intensity of renal surveillance, baseline renal function, patient selection, and procedural characteristics limit direct comparison across studies. A detailed summary of renal outcomes, AKI definitions, contrast exposure, and associated clinical findings across the included studies is presented in Table 2.

### 3.4. Risk Factors for AKI

Pre-existing renal dysfunction was the most consistently identified patient-related risk factor for AKI. Nombela-Franco et al. found that lower baseline eGFR independently predicted AKI, with an OR of 1.32 for each 10 mL/min decrease in glomerular filtration rate (95% CI 1.09–1.61; *p* = 0.004) [12].

In the study by Sedaghat et al., baseline renal function was numerically poorer among patients who developed AKI, but the difference was not statistically significant. A higher contrast volume-to-GFR ratio showed a nonsignificant trend toward increased AKI risk (OR 1.24 per unit increase; 95% CI 0.97–1.58; *p* = 0.08), whereas absolute contrast volume did not differ significantly between patients with and without AKI [13]. Accordingly, this study did not establish contrast exposure as an independent determinant of AKI.

In the administrative cohort reported by Nazir et al., CKD, non-elective admission, coagulopathy, weight loss, prior coronary artery disease, heart failure, diabetes mellitus, and anemia were independently associated with an increased risk of coded AKI [14]. Zhang et al. identified lower BMI, hypertension, and advanced CKD stages 4–5 as independent predictors of AKI [15].

Collectively, these findings identify impaired baseline renal function and broader comorbidity burden as the most consistent determinants of renal vulnerability. In contrast, evidence that procedural contrast exposure independently predicts AKI after LAAO remains limited and inconclusive.

### 3.5. Clinical and Prognostic Impact of AKI

AKI following LAAO was consistently associated with adverse clinical outcomes in studies evaluating its prognostic significance. Nombela-Franco et al. reported higher mortality among patients who developed AKI and identified AKI as an independent predictor of mid-term mortality (HR 2.00; 95% CI 1.02–3.91; *p* = 0.044) over a median follow-up of 18 months [12]. Sedaghat et al. similarly reported higher mortality at 6 months and 1 year among patients developing AKI [13].

In the largest included cohort, Nazir et al. found that coded AKI was strongly associated with adverse in-hospital outcomes, including in-hospital mortality (aOR 16.01; 95% CI 8.48–30.21), stroke or transient ischemic attack (aOR 2.50; 95% CI 1.69–3.70), systemic embolization (aOR 3.78; 95% CI 1.64–8.70), bleeding or transfusion (aOR 1.96; 95% CI 1.50–2.56), vascular complications (aOR 3.53; 95% CI 1.94–6.42), and pericardial tamponade requiring intervention (aOR 6.83; 95% CI 4.37–10.66) [14]. AKI was also associated with increased 180-day all-cause readmission (aOR 1.43; 95% CI 1.09–1.88). Zhang et al. further reported associations between post-LAAC AKI and long-term mortality and MACE during follow-up [15].

Overall, AKI was consistently associated with increased morbidity and mortality across the available studies. However, because the evidence was observational, these associations should not be interpreted as demonstrating that AKI itself directly caused the subsequent adverse clinical events.

### 3.6. Procedural Feasibility and Safety of Contrast-Sparing and Zero-Contrast LAAO

Fourteen studies evaluated procedural strategies aimed at reducing or eliminating the use of iodinated contrast during LAAO. Early feasibility studies consistently demonstrated that LAAO could be performed with little or no iodinated contrast using echocardiographic guidance. Wisst et al. [24] compared contrast-free with conventional Amulet implantation in 46 patients and reported 100% procedural success in both groups. Sedaghat et al. [16] similarly reported successful contrast-free Amulet implantation in all patients in a propensity-matched comparison with conventional LAAO. Saad Shaukat et al. [25] demonstrated successful TEE-guided WATCHMAN implantation without iodinated contrast in 12 patients with advanced CKD or contrast allergy. Chu et al. [26] reported successful ICE- and electroanatomic mapping-guided LAAO without fluoroscopy or iodinated contrast in all seven treated patients, with procedural outcomes comparable with those of seven propensity-matched conventional controls. Flautt et al. [27] subsequently reported successful contrast-free ICE-guided LAAC in 53 patients, including 41 procedures performed without fluoroscopy.

Additional studies extended these approaches to patients considered at increased risk from contrast exposure. Zou et al. [28] evaluated a TEE-only non-fluoroscopic strategy in 32 patients, most of whom had impaired baseline renal function, and reported procedural outcomes comparable with those of a fluoroscopy-guided cohort after propensity matching. Chakravarty et al. [29] described successful TEE-guided zero-contrast WATCHMAN FLX implantation in 110 consecutive patients, whereas Blusztein et al. [17] reported successful zero-contrast LAAO in 31 patients using hybrid echocardiography–fluoroscopy guidance. In patients with advanced CKD, Fukushima et al. [18] achieved successful implantation in all 15 patients; complete contrast avoidance was achieved in 13 patients, whereas two required small contrast volumes of 6 and 15 mL. Okada et al. [23] likewise reported successful contrast-free WATCHMAN FLX implantation in all 10 patients with CKD.

Larger contemporary series further supported procedural feasibility. Vij et al. [19] reported a device success rate of 97.5% in 118 patients undergoing contrast-free LAMBRE LAAO, whereas Ateş et al. [20] reported a procedural success rate of 99.7% among 324 consecutive zero-contrast procedures. More recent comparative observational studies by Cieślik et al. [30] and Demian et al. [21] reported broadly comparable procedural safety and efficacy between zero-contrast and conventional contrast-guided approaches. Collectively, these studies support the technical feasibility of contrast-sparing and zero-contrast LAAO in selected patients. However, most studies were observational, several were small or uncontrolled, and procedural success should not be interpreted as evidence of renal protection.

### 3.7. Renal Outcomes Reported in Contrast-Sparing and Zero-Contrast Studies

Renal outcomes were inconsistently assessed across the 14 contrast-sparing or zero-contrast studies. Only a minority systematically reported AKI or postprocedural renal-function changes. Chakravarty et al. [29] reported transient AKI in 6 of 110 patients (5.5%) despite complete avoidance of iodinated contrast; none required hemodialysis. Vij et al. [19] reported no AKI in their contrast-free LAMBRE cohort, whereas Ateş et al. [20] reported no AKI according to KDIGO criteria during the index hospitalization and stable renal function in the CKD subgroup. These findings describe renal outcomes within individual cohorts but, in the absence of an appropriate contemporaneous comparator, do not demonstrate that contrast avoidance reduces the risk of AKI.

Other studies reported broader renal outcomes rather than standardized AKI ascertainment. Fukushima et al. [18] reported no acute kidney disease during 45-day follow-up, whereas Okada et al. [23] observed no significant deterioration in postprocedural eGFR. These outcomes were retained using the terminology of the original studies and were not considered interchangeable with creatinine-based AKI defined according to standardized criteria.

In contrast, AKI and postprocedural renal-function changes were not separately reported in the studies by Wisst et al. [24], Sedaghat et al. [16], Saad Shaukat et al. [25], Chu et al. [26], Flautt et al. [27], Blusztein et al. [17], Zou et al. [28], Cieślik et al. [30], and Demian et al. [21]. These studies therefore contributed evidence regarding procedural feasibility and safety but not regarding prevention of AKI. Importantly, absence of a reported AKI endpoint was not interpreted as absence of AKI events.

Overall, the available evidence does not establish iodinated contrast exposure as the sole or independent determinant of post-LAAO AKI. The occurrence of AKI despite complete contrast avoidance, together with the heterogeneous renal risk profiles of the included populations, supports a multifactorial mechanism involving baseline renal dysfunction, hemodynamic factors, comorbidity burden, and other periprocedural influences. Accordingly, current evidence supports the feasibility of contrast-sparing approaches but remains insufficient to establish a nephroprotective effect.

**Figure 2:**
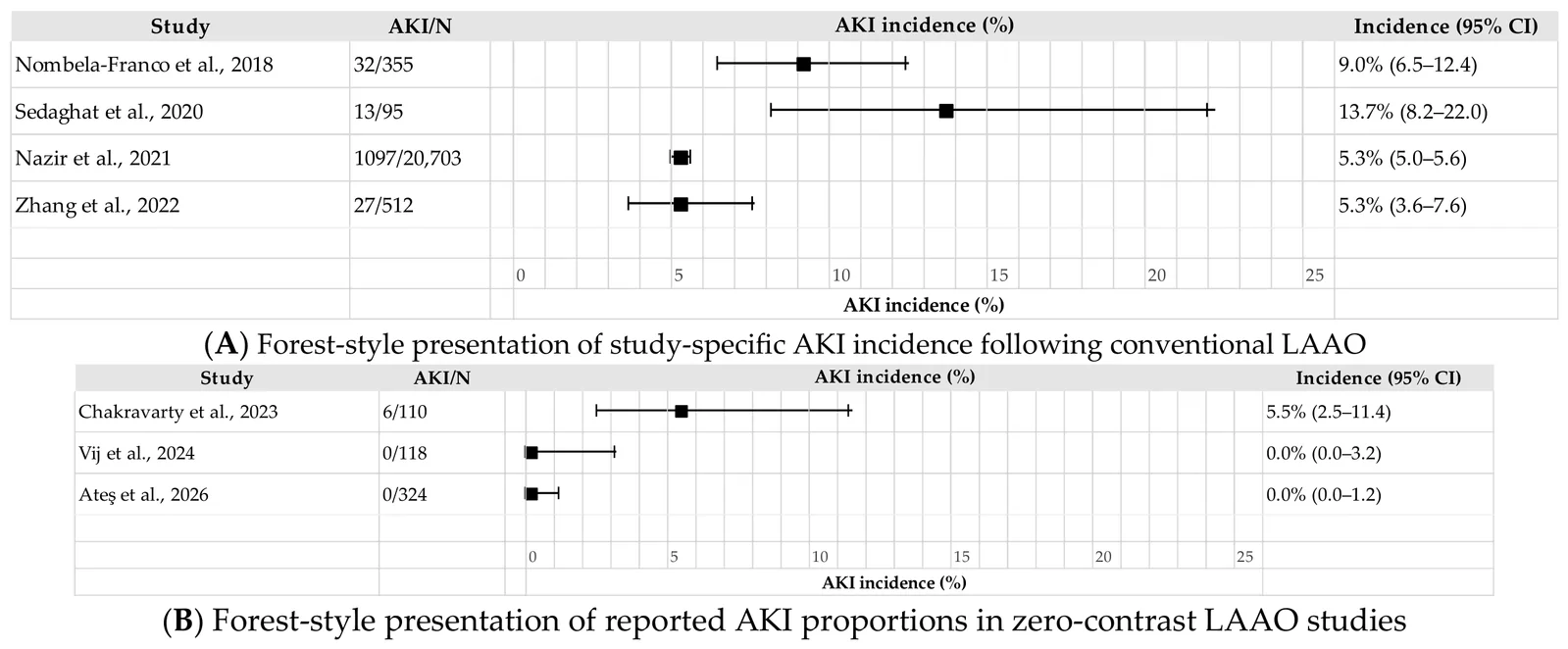
Forest-style presentation of study-specific AKI outcomes with 95% confidence intervals. (**A**) Conventional LAAO studies. (**B**) Zero-contrast LAAO studies with extractable AKI event counts and denominators. Confidence intervals were calculated using the Wilson score method. No pooled estimate was calculated. Because AKI definitions and ascertainment methods differed across studies, the estimates are descriptive and should not be interpreted as directly comparable. In Nazir et al., AKI was identified using administrative diagnostic coding; renal outcome ascertainment was not standardized across all zero-contrast studies. Abbreviations: AKI, acute kidney injury; LAAO, left atrial appendage occlusion; CI, confidence interval [12,13,14,15,19,20,29].

To facilitate visual comparison across studies, study-specific AKI estimates with corresponding 95% confidence intervals are presented in Figure 2, with conventional and zero-contrast LAAO studies shown separately. No pooled estimate was calculated.

### 3.8. Risk of Bias

Methodological quality and risk of bias were assessed using design-specific JBI critical appraisal tools. The 18 included studies comprised cohort and comparative observational studies as well as uncontrolled case series, and item-level judgments are presented separately in Supplementary Table S2A,B. No aggregate numerical quality score was calculated.

Across the cohort and comparative observational studies, recurrent methodological limitations included nonrandomized treatment allocation, potential residual confounding, incomplete or differential follow-up, and, in some studies, selection of participants according to the availability of renal or procedural data. Propensity-matched comparative studies improved baseline comparability but remained susceptible to residual confounding and selection bias. Large registry-based analyses provided greater statistical precision; however, the administrative database study by Nazir et al. relied on diagnostic coding rather than laboratory-based AKI ascertainment, and the timing of AKI relative to LAAO could not be established. In contrast, studies using creatinine-based AKIN or KDIGO criteria provided more direct renal outcome ascertainment but were generally smaller and included relatively few AKI events.

The uncontrolled case-series evidence was primarily informative regarding procedural feasibility and safety. Several studies clearly described the procedural strategy and clinical outcomes, but small sample sizes, single-center designs, absence of contemporaneous contrast-guided comparators, and incompletely reported enrollment or follow-up procedures limited causal interpretation and generalizability. In particular, several contrast-sparing or zero-contrast studies did not systematically define or ascertain AKI, and therefore could not establish a nephroprotective effect despite high procedural success.

Overall, the most consistent evidence concerned the association between AKI and adverse clinical outcomes, supported across several observational cohorts, including studies using standardized creatinine-based definitions. Evidence regarding AKI incidence and the role of baseline renal dysfunction was also relatively consistent, although direct comparability was limited by differences in AKI definitions, assessment windows, patient selection, and outcome ascertainment. In contrast, evidence supporting a nephroprotective effect of contrast-sparing or zero-contrast LAAO was substantially more limited. Most studies were uncontrolled observational series, several did not systematically assess or separately report AKI, and available comparative studies were not designed to establish renal protection. Accordingly, conclusions regarding prevention of AKI through contrast avoidance should be considered hypothesis-generating rather than definitive.

## 4. Discussion

This systematic review provides an integrated assessment of AKI following percutaneous LAAO, encompassing its incidence, clinical determinants, prognostic implications, and the emerging role of contrast-sparing and zero-contrast procedural strategies. Four principal observations arise from the available evidence. First, AKI is a clinically relevant complication of conventional LAAO, with reported incidences ranging from approximately 5.3% to 13.7%. Second, pre-existing renal dysfunction consistently emerges as an important marker of susceptibility, whereas the independent contribution of procedural iodinated contrast exposure remains uncertain. Third, development of AKI is consistently associated with adverse short- and long-term outcomes. Finally, contemporary experience demonstrates that LAAO can increasingly be performed with minimal or no iodinated contrast, although current evidence does not establish that contrast elimination itself prevents AKI. The clinical relevance of these findings should be considered in the context of the expanding role of LAAO in contemporary stroke prevention. Randomized evidence from PROTECT AF and PREVAIL established percutaneous LAA closure as an alternative to warfarin in selected patients with non-valvular AF [31,32], while the PRAGUE-17 trial subsequently demonstrated that LAAO was non-inferior to direct oral anticoagulants for major cardiovascular, neurological, and bleeding outcomes in high-risk patients [33]. Real-world evidence from the EWOLUTION and NCDR LAAO registries further demonstrated that LAAO is increasingly performed in older, highly comorbid populations with substantial thromboembolic and bleeding risk [34,35,36]. Contemporary guidelines and expert consensus statements consequently recognize percutaneous LAAO as an established stroke-prevention option in appropriately selected patients with AF who are unsuitable for long-term OAC [1,37,38]. Renal dysfunction is highly relevant in this population because CKD simultaneously increases thromboembolic and bleeding risk and may complicate pharmacological stroke-prevention strategies. Previous observational studies and registry analyses have demonstrated that LAAO is technically feasible across the spectrum of renal dysfunction, including advanced CKD and ESRD, although impaired renal function remains associated with a greater burden of cardiovascular comorbidity and worse long-term prognosis [5,6,7,8,39,40]. In the German multicenter LAARGE registry, implantation success remained high across renal-function strata despite a greater cardiovascular risk profile among patients with CKD [39]. Contemporary meta-analyses have similarly supported the procedural feasibility of LAAO in CKD while demonstrating that patients with CKD or ESRD remain at intrinsically higher clinical risk [10,41].

### 4.1. Incidence and Mechanisms of AKI Following LAAO

The available evidence indicates that AKI is a clinically relevant event in patients undergoing LAAO, but its reported frequency depends on how and when renal injury is assessed. The three creatinine-based cohorts provide more direct evidence of postprocedural AKI than the administrative cohort because they compare renal function before and after the procedure [12,13,15]. Nevertheless, their estimates remain sensitive to differences in diagnostic criteria, assessment windows, patient selection, and completeness of surveillance. A 48-h assessment and a 7-day assessment may capture different events, while AKI occurring after discharge may be missed.

The administrative study provides complementary evidence on the association between coded AKI and adverse hospital outcomes [14]. However, the absence of laboratory data and uncertainty about whether AKI preceded or followed LAAO prevent its use as a directly comparable estimate of new postprocedural AKI. Standardized prospective studies should specify baseline creatinine, diagnostic criteria, measurement schedules, and postdischarge surveillance, while distinguishing incident AKI from kidney injury already present before LAAO.

The mechanisms underlying AKI after LAAO are unlikely to be explained by iodinated contrast exposure alone. Patients undergoing LAAO frequently present with advanced age, CKD, heart failure, diabetes, anemia, vascular disease, and other conditions associated with reduced renal reserve. Periprocedural hypotension, changes in intravascular volume, anesthesia-related hemodynamic alterations, bleeding, inflammatory responses, and exposure to nephrotoxic medications may act individually or synergistically. Accordingly, renal injury occurring after a contrast-guided procedure should not automatically be attributed to contrast administration. This distinction is supported by the broader contrast-associated AKI literature. A systematic review of controlled studies found similar rates of AKI among patients undergoing contrast-enhanced and non-contrast imaging, emphasizing the potential importance of confounding by underlying illness [42,43]. More recently, consensus statements from the American College of Radiology and the National Kidney Foundation formally distinguished contrast-associated AKI (CA-AKI), which describes AKI occurring temporally after contrast exposure, from contrast-induced AKI (CI-AKI), which requires a causal relationship with iodinated contrast [44]. This conceptual distinction is particularly relevant to LAAO and supports a multifactorial rather than exclusively contrast-mediated model of postprocedural renal injury.

### 4.2. Renal Vulnerability and Procedural Risk

Baseline renal dysfunction emerged as the most consistent marker of susceptibility to AKI across the included studies. Nombela-Franco et al. demonstrated that lower baseline eGFR was independently associated with subsequent AKI [12]. In contrast, Sedaghat et al. observed numerically poorer baseline renal function among patients who developed AKI and a trend toward increased AKI risk with a higher contrast volume-to-GFR ratio; however, these associations did not reach statistical significance, and absolute contrast volume was not significantly different between patients with and without AKI [13]. Zhang et al. similarly identified advanced CKD stages 4–5 as an independent predictor of AKI, together with hypertension and lower body mass index [15].

These findings suggest that renal vulnerability after LAAO is more strongly related to the underlying renal and clinical substrate than to contrast exposure alone. Procedural contrast burden may remain relevant in selected high-risk patients, particularly when renal reserve is limited, but the available evidence does not establish iodinated contrast as an independent causal determinant of post-LAAO AKI. The occurrence of AKI despite completely zero-contrast implantation further supports a multifactorial model in which baseline CKD, hemodynamic instability, volume status, bleeding, anemia, heart failure, diabetes, vascular disease, nephrotoxic medications, and other periprocedural factors may contribute to renal injury.

Renal dysfunction in patients with atrial fibrillation should therefore be interpreted within the broader cardiovascular–kidney–metabolic burden rather than as an isolated determinant of procedural risk. CKD frequently coexists with heart failure, diabetes, hypertension, vascular disease, anemia, and frailty, all of which may influence both periprocedural vulnerability and long-term prognosis. Recent prospective registry data further support this broader perspective, showing that a higher cardiovascular–kidney–metabolic risk burden in patients with atrial fibrillation was associated with increased all-cause mortality and major adverse cardiovascular events [45].

Accordingly, renal impairment alone should not be considered sufficient to favor LAAO over pharmacological stroke-prevention strategies. The choice between LAAO and oral anticoagulation should remain individualized according to renal function, bleeding risk, thromboembolic risk, comorbidity burden, suitability for long-term anticoagulation, and the overall clinical phenotype. In kidney-transplant recipients with atrial fibrillation, a recent propensity-matched real-world analysis found direct oral anticoagulant therapy to be associated with a lower risk of the composite of all-cause death, thromboembolic events, and major bleeding compared with vitamin K antagonists [46]. These findings should not be interpreted as direct evidence against LAAO, but rather as complementary evidence supporting individualized selection between pharmacological and interventional stroke-prevention strategies in patients with complex renal disease.

Overall, baseline renal dysfunction should be regarded as one component of a broader risk phenotype rather than an isolated indication for a specific stroke-prevention strategy. Renal risk assessment before LAAO should therefore incorporate kidney function together with cardiovascular comorbidities, hemodynamic status, bleeding risk, procedural characteristics, and the anticipated feasibility of long-term pharmacological therapy.

### 4.3. AKI as a Prognostic Marker After LAAO

A particularly consistent finding of this review was the association between AKI and adverse clinical outcomes. Nombela-Franco et al. demonstrated an independent association between AKI and mid-term mortality, while Sedaghat et al. reported higher mortality at both 6 months and 1 year among patients developing AKI. The large analysis by Nazir et al. reinforced these observations, demonstrating a strong association between AKI and in-hospital mortality as well as stroke or transient ischemic attack, systemic embolism, bleeding, vascular complications, tamponade, prolonged hospitalization, and subsequent readmission [12,13,14]. In the latter cohort, the adjusted odds ratio for in-hospital mortality was 16.01 among patients with AKI. Zhang et al. further extended this association to long-term mortality and MACE during follow-up [15]. These associations should not necessarily be interpreted as demonstrating that AKI directly causes all subsequent adverse events. AKI may instead function partly as an integrated marker of procedural stress, hemodynamic instability, underlying CKD, and overall comorbidity burden. Nevertheless, the broader nephrology literature confirms that even an episode of AKI is associated with increased subsequent mortality and long-term renal morbidity [47]. Thus, the consistent prognostic association observed following LAAO supports routine attention to postprocedural renal function rather than considering transient creatinine elevation an incidental laboratory finding. From a clinical perspective, this observation shifts the emphasis from management of established AKI toward preprocedural identification of vulnerable patients. Baseline renal function, volume status, potentially nephrotoxic medications, previous AKI, anticipated contrast requirements, and hemodynamic vulnerability should therefore form part of individualized procedural planning.

### 4.4. Contrast-Sparing and Zero-Contrast LAAO: Procedural Feasibility and Renal Evidence

The evidence supporting the procedural feasibility of contrast-sparing and zero-contrast LAAO should be distinguished from evidence regarding their effects on renal outcomes. Contemporary imaging and device technologies provide a procedural basis for reducing reliance on iodinated contrast [37,48,49]. Studies using different devices and imaging approaches have reported high implantation success rates, including in selected patients with CKD [16,17,18,19,20,23,29]. Previous systematic evidence has also supported the feasibility of zero-contrast implantation [50]. Comparative observational studies by Cieślik et al. and Demian et al. reported similar procedural outcomes between zero-contrast and contrast-guided approaches [21,30]. However, nonrandomized treatment allocation, operator experience, and patient selection limit conclusions regarding comparative safety and generalizability.

The evidence for renal protection is substantially more limited. Several procedural studies did not separately report AKI and therefore cannot establish whether contrast avoidance reduces its incidence [16,17,21,25]. Studies reporting renal outcomes used heterogeneous endpoints, including AKI, changes in eGFR, and acute kidney disease [18,19,20,23,29]. These outcomes should not be treated as interchangeable. Furthermore, the absence of AKI in an uncontrolled cohort cannot establish a preventive effect without an appropriate comparator and comparable renal surveillance. Stable postprocedural eGFR likewise does not, by itself, demonstrate that a strategy prevents AKI.

Chakravarty et al. reported transient AKI in 6 of 110 patients despite zero-contrast implantation [29], whereas Ateş et al. reported no KDIGO-defined AKI during the index hospitalization in their cohort [20]. These observations describe outcomes in different populations and should not be interpreted as comparative estimates of nephroprotective effectiveness. AKI occurring despite contrast avoidance is consistent with contributions from factors other than iodinated contrast, but does not exclude the possibility that contrast reduction could benefit some patients.

Accordingly, current evidence supports contrast-sparing and zero-contrast LAAO as feasible procedural options in selected patients. It does not establish that these approaches prevent AKI. Their potential renal benefit remains a hypothesis requiring prospective comparative evaluation with standardized AKI definitions, consistent assessment schedules, and adequate control of baseline renal risk and other confounding factors.

### 4.5. Clinical Implications and a Proposed Renal-Tailored Framework

The findings of this review support individualized procedural planning in patients undergoing LAAO, particularly when renal vulnerability is present. Such planning may include assessment of baseline serum creatinine and eGFR, volume status, previous AKI, potentially nephrotoxic medications, and hemodynamic vulnerability. The choice of procedural imaging and contrast strategy should additionally consider patient anatomy, imaging quality, procedural complexity, and operator experience.

Contrast minimization or zero-contrast implantation may be considered when technically appropriate, particularly in patients in whom iodinated contrast exposure is undesirable. However, the available evidence does not establish that contrast avoidance reduces the incidence of AKI, nor does it identify a specific subgroup in whom zero-contrast LAAO provides a proven renal benefit. Postprocedural renal surveillance therefore remains relevant even after completely contrast-free implantation.

Evidence supporting other renal preventive measures largely originates from coronary angiography and cardiac catheterization populations and should not be directly extrapolated to LAAO. Hemodynamic-guided fluid administration reduced contrast-associated AKI in the POSEIDON trial [51], whereas the PRESERVE trial did not demonstrate benefit from sodium bicarbonate over sodium chloride or from acetylcysteine over placebo [52]. These findings provide context for individualized volume management but do not establish efficacy in the LAAO population, particularly in patients with heart failure or susceptibility to volume overload.

Accordingly, renal-tailored LAAO should be regarded as a pragmatic, hypothesis-generating framework rather than an evidence-validated nephroprotective strategy. It may integrate procedural imaging, minimization of unnecessary contrast exposure, hemodynamic management, medication review, and postprocedural renal surveillance according to individual patient risk. Its effectiveness in reducing AKI and improving subsequent clinical outcomes requires prospective comparative evaluation using standardized renal endpoints.

### 4.6. Evidence Gaps and Future Research

Despite the expanding literature, several important evidence gaps remain. First, no adequately powered randomized trial has tested whether contrast minimization or complete contrast elimination reduces the incidence of AKI after LAAO. Second, renal endpoints remain inconsistently defined, with studies using KDIGO criteria, alternative creatinine-based definitions, administrative coding, changes in eGFR, or no explicit AKI definition. Third, most zero-contrast studies are observational and susceptible to selection bias because operators may preferentially select patients with favorable anatomy or procedures considered technically suitable for contrast avoidance. Future studies should prospectively apply standardized AKI definitions [53], report baseline and serial postprocedural serum creatinine and eGFR, quantify contrast exposure, and systematically capture hemodynamic and procedural variables. Importantly, future studies should distinguish CA-AKI from CI-AKI, consistent with contemporary consensus terminology, and avoid automatically assigning causality to contrast solely on the basis of temporal association [42,44]. The renal benefit of zero-contrast LAAO should ideally be evaluated in prospective comparative studies enriched for patients at high renal risk, particularly those with advanced CKD, previous AKI, or multiple recognized predictors of peri-interventional renal injury. The rapid transition from small feasibility series to large real-world cohorts and direct comparative studies [20,21] indicates that such evaluation is now technically feasible. Future research should also determine whether specific device platforms or imaging strategies facilitate contrast minimization without compromising procedural safety. Contemporary LAAO technology has demonstrated high efficacy across WATCHMAN FLX and Amulet platforms [48,49], and accumulated randomized and real-world experience demonstrates that LAAO outcomes continue to improve with device evolution and operator experience [33,34]. Whether these advances can translate into measurable renal benefit through reduced contrast dependence remains an important unanswered question.

### 4.7. Strengths and Limitations

The principal strength of this systematic review is its integrated evaluation of two previously fragmented areas of evidence: AKI occurring after conventional LAAO and the rapidly developing literature on contrast-sparing and zero-contrast implantation. Rather than limiting the analysis to patients with pre-existing CKD, the review evaluates AKI as a procedural complication and examines its incidence, determinants, prognostic implications, and the evidence concerning contrast-sparing and zero-contrast procedural strategies within a unified framework. A further methodological strength is the use of a structured systematic-review framework. The review was conducted and reported according to PRISMA 2020 recommendations [22], while methodological appraisal was performed using design-specific JBI approaches. The JBI case-series appraisal framework was specifically developed to evaluate methodological quality and potential bias in uncontrolled case-series evidence [54], whereas the revised JBI cohort tool provides a contemporary domain-based framework for assessing bias related to exposure classification, confounding, outcome assessment, participant retention, and statistical validity in observational cohort studies [55]. This distinction was particularly relevant because the present evidence base contains both relatively large observational cohorts and small uncontrolled zero-contrast series. Several limitations should nevertheless be acknowledged. The included studies were predominantly observational and heterogeneous in design, population, device type, baseline renal function, procedural technique, AKI definition, and duration of follow-up. Several zero-contrast studies were small, uncontrolled case series, while the largest AKI analysis relied on administrative coding. Renal outcomes were not consistently reported in studies primarily evaluating procedural feasibility, and the absence of standardized AKI definitions limited direct comparison between studies. Furthermore, the clinical and methodological heterogeneity of the available evidence precluded meaningful quantitative pooling. Finally, the observational nature of the available evidence prevents causal inference regarding either the contribution of iodinated contrast to post-LAAO AKI or the renal protective effect of contrast avoidance.

## 5. Conclusions

AKI is a clinically relevant complication following percutaneous LAAO and is consistently associated with adverse clinical outcomes. Pre-existing renal dysfunction identifies a particularly vulnerable population, although postprocedural renal injury appears to reflect a multifactorial process involving baseline kidney function, comorbidity burden, hemodynamic and procedural factors, and potentially iodinated contrast exposure.

Evidence concerning the incidence, predictors, and prognosis of AKI should be distinguished from evidence supporting the procedural feasibility of contrast-sparing or zero-contrast LAAO. Contemporary studies demonstrate that contrast minimization and complete contrast avoidance are technically feasible across different devices and imaging strategies, including in patients with impaired renal function. However, renal outcomes were inconsistently assessed, several feasibility studies did not systematically report AKI, and direct comparative renal evidence remains limited. Consequently, current evidence does not establish that contrast avoidance prevents AKI or provides a proven nephroprotective benefit.

A renal-tailored approach to LAAO may provide a pragmatic framework for individualized procedural planning, integrating baseline renal risk, comorbidities, imaging strategy, contrast exposure, hemodynamic management, medication review, and postprocedural renal surveillance. This framework should be regarded as hypothesis-generating rather than evidence-validated. Prospective comparative studies using standardized AKI definitions, prespecified renal assessment schedules, and adequate adjustment for baseline renal and clinical risk are needed to determine whether contrast-sparing strategies improve renal and clinical outcomes.

## Figures and Tables

**Table 1:** Characteristics of the 18 included studies.

Study	Year	Design	N	Intervention/Population	Renal/Clinical Focus	Key Finding Relevant to the Review
A. Studies primarily evaluating AKI after LAAO						
Nombela-Franco et al. [12]	2018	Multicenter observational registry	355	Percutaneous LAAC	AKI incidence, predictors, and prognosis	AKI occurred in 32/355 patients (9.0%); lower baseline eGFR independently predicted AKI; AKI independently predicted mid-term mortality.
Sedaghat et al. [13]	2020	Multicenter retrospective observational study	95	LAAC with Amplatzer occluders	AKI incidence, predictors, and clinical relevance	AKI occurred in 13/95 patients (13.7%). A higher contrast volume/GFR ratio showed a nonsignificant trend toward increased AKI risk (*p* = 0.08). AKI was associated with higher mortality at 6 months and 1 year.
Nazir et al. [14]	2021	National database retrospective cohort	20,703 (weighted national estimate)	Percutaneous LAAC	AKI risk factors and in-hospital/180-day outcomes	AKI was coded in 5.3% of index hospitalizations and was associated with higher in-hospital mortality, complications, and readmission. Its timing relative to LAAO could not be determined, limiting interpretation as postprocedural AKI incidence.
Zhang et al. [15]	2022	Single-center retrospective cohort	512	Percutaneous LAAC	AKI incidence, predictors, and 3-year outcomes	AKI occurred in 27/512 patients (5.3%); CKD stages 4–5, hypertension, and lower BMI independently predicted AKI; AKI was associated with MACE and mortality.
B. Studies evaluating contrast-sparing or zero-contrast LAAO						
Wisst et al. [24]	2018	Prospective single-center registry	46 (21 no-contrast vs. 25 contrast-guided)	Contrast-free vs. conventional Amulet LAAC	Procedural feasibility and safety; renal relevance	Contrast-free LAAC was successfully performed without iodinated contrast; procedural success was 100% in both groups. Contrast use was 0 mL in the no-contrast group versus 76.2 ± 49.7 mL in the conventional group. AKI and postprocedural renal function were not systematically assessed.
Sedaghat et al. [16]	2019	Propensity-matched observational study	40 (20 vs. 20)	Contrast-free vs. conventional Amulet LAAO	Procedural feasibility and safety	Procedural success was 100% in both groups; contrast-free LAAO was associated with lower radiation exposure and shorter fluoroscopy time. AKI was not separately reported.
Saad Shaukat et al. [25]	2020	Retrospective single-center case series	12	TEE-guided WATCHMAN implantation without iodinated contrast in patients with advanced CKD or contrast allergy	Procedural feasibility and safety in patients at risk from contrast exposure	WATCHMAN implantation without iodinated contrast was successful in all 12 patients. Contrast avoidance was prompted by CKD stage IV in 5 patients, rapidly progressive CKD stage III in 1, and contrast allergy in 6. No immediate or delayed procedural complications were reported through 45 days; AKI was not separately assessed.
Chu et al. [26]	2020	Comparative observational propensity-matched study	14 (7 zero-fluoroscopy/zero-contrast vs. 7 conventional)	ICE- and electroanatomic mapping-guided LAMBRE LAAO without fluoroscopy or iodinated contrast vs. conventional LAAO	Procedural feasibility and safety of zero-contrast implantation	Zero-fluoroscopy, zero-contrast LAAO was successfully performed in all 7 patients, with 100% acute complete closure and no procedural complications. Outcomes were comparable with the propensity-matched conventional group. AKI and postprocedural renal function were not separately assessed.
Flautt et al. [27]	2022	Single-center observational case series/research correspondence	53	ICE-guided LAAC without angiographic contrast; fluoroscopy-free strategy in 41/53 patients	Procedural feasibility and safety of contrast-free LAAC	No iodinated contrast was used in any of the 53 procedures. LAAC was successful in all patients without procedural complications or acute peridevice leak. In 41 patients planned for fluoroscopy-free implantation, no bailout fluoroscopy was required. AKI and postprocedural renal function were not separately assessed.
Zou et al. [28]	2023	Retrospective single-center propensity-matched cohort study	126 (32 TEE-only vs. 94 fluoroscopy-guided; propensity-matched 25 vs. 25)	TEE-guided non-fluoroscopic LAAC in patients at high risk from fluoroscopy/contrast exposure vs. conventional fluoroscopy-guided LAAC	Procedural feasibility and safety in patients at increased contrast-related risk	LAAC was successfully performed in all patients. The TEE-only group had lower baseline renal function (mean eGFR 52.7 ± 8.8 mL/min; 78.1% had eGFR <60 mL/min) and achieved procedural outcomes comparable with the fluoroscopy-guided group after propensity matching. AKI and postprocedural renal function were not separately assessed.
Chakravarty et al. [29]	2023	Single-center consecutive case series (Research Letter)	110	TEE-guided zero-contrast WATCHMAN FLX LAAO	Procedural feasibility and safety; renal outcomes	Zero-contrast LAAO was successfully performed in 110 consecutive patients; transient AKI occurred in 6 patients (5.5%), with no requirement for hemodialysis.
Blusztein et al. [17]	2023	Single-center registry/case series	31	Zero-contrast LAAO using hybrid echocardiography–fluoroscopy guidance	Procedural feasibility and safety	Procedural success was 100%; no iodinated contrast was used and no device-related complications were reported at 45 days. AKI was not separately reported.
Fukushima et al. [18]	2024	Single-center retrospective case series	15	Attempted zero-contrast LAAC in CKD stages 3b–5	Renal outcomes; procedural feasibility and safety	Procedural success was 100%; complete contrast avoidance was achieved in 13/15 patients, while 2 required 6 and 15 mL of contrast. No periprocedural complications or acute kidney disease were reported during 45-day follow-up.
Okada et al. [23]	2024	Single-center retrospective case series	10	Contrast-free WATCHMAN FLX LAAC in patients with CKD	Renal function; procedural feasibility and safety	Contrast-free implantation was successful in all patients; postprocedural eGFR did not significantly change from baseline and no in-hospital adverse events were reported.
Vij et al. [19]	2024	Multicenter retrospective registry/case series	118	Contrast-free LAMBRE LAAC	Procedural safety; renal outcomes	Device success was 97.5%; no AKI was reported. Mean eGFR was approximately 50 mL/min, and 5 patients were receiving hemodialysis.
Ateş et al. [20]	2026	Single-center retrospective consecutive cohort	324	Zero-contrast LAAC	Procedural safety and efficacy; renal outcomes	Procedural success was 99.7%; no AKI according to KDIGO criteria occurred during the index hospitalization; renal function remained stable in the CKD subgroup.
Cieślik et al. [30]	2026	Nonrandomized comparative observational study	159 (84 vs. 75)	Zero-contrast vs. contrast-guided LAAO	Procedural safety and efficacy	Similar procedural safety and efficacy outcomes were reported between groups. AKI was not separately reported as a renal endpoint.
Demian et al. [21]	2026	Single-center comparative registry	621 (326 vs. 295)	Zero-contrast vs. contrast-guided WATCHMAN FLX LAAO	Procedural and long-term outcomes	Similar procedural success and adverse-event rates were reported between strategies; 45-day and 1-year outcomes were assessed. AKI was not separately reported.

Note: Studies were grouped according to their primary focus. Renal outcomes reported in Group B were interpreted separately from procedural outcomes. Procedural feasibility and safety findings, and renal outcomes from uncontrolled cohorts, were not considered sufficient to establish nephroprotection. “AKI not separately reported” does not indicate the absence of AKI events. Abbreviations: AKI, acute kidney injury; BMI, body mass index; CKD, chronic kidney disease; eGFR, estimated glomerular filtration rate; KDIGO, Kidney Disease: Improving Global Outcomes; LAAC, left atrial appendage closure; LAAO, left atrial appendage occlusion; MACE, major adverse cardiovascular events; TEE, transesophageal echocardiography.

**Table 2:** Renal outcomes, acute kidney injury definitions, contrast exposure, and clinical findings across the included studies.

Study	N	Renal Status/AKI Definition	Contrast Strategy	AKI/Renal Outcome	Main Predictors or Clinical Findings	Follow-Up
A. Studies primarily evaluating AKI after LAAO						
Nombela-Franco et al., 2018 [12]	355	AKI defined using AKIN criteria as an increase in SCr ≥0.3 mg/dL or ≥50% from baseline within 48 h, or need for hemodialysis during the index hospitalization. SCr was assessed at baseline and serially after LAAO; patients receiving chronic dialysis were excluded.	Conventional contrast-guided LAAC; median contrast volume 150 mL (IQR 102–228). Contrast volume did not differ significantly between patients with and without AKI.	AKI in 32/355 patients (9.0%).	Lower baseline eGFR independently predicted AKI (OR 1.32 per 10 mL/min decrease; 95% CI 1.09–1.61; *p* = 0.004). AKI independently predicted mid-term mortality (HR 2.00; 95% CI 1.02–3.91; *p* = 0.044).	Creatinine-based AKI assessed within 48 h; median clinical follow-up 18 months.
Sedaghat et al., 2020 [13]	95	AKI defined using serum creatinine-based AKIN criteria, comparing baseline creatinine with the peak value within 48 h after LAAO; urine-output criteria were not used. Patients receiving chronic dialysis were excluded.	Conventional contrast-guided LAAC with Amplatzer occluders. Contrast volume was 127 ± 83 mL in patients with AKI versus 109 ± 92 mL in those without AKI (*p* = 0.41).	AKI in 13/95 patients (13.7%).	Baseline renal function was numerically poorer in patients with AKI but the difference was not statistically significant. A higher contrast volume/GFR ratio showed a nonsignificant trend toward increased AKI risk (OR 1.24 per unit increase; 95% CI 0.97–1.58; *p* = 0.08); absolute contrast volume was not associated with AKI. AKI was associated with higher mortality at 6 months and 1 year.	AKI assessed within 48 h after LAAO; clinical follow-up up to 1 year.
Nazir et al., 2021 [14]	20,703 (weighted national estimate)	AKI identified using ICD-10-CM diagnostic codes N17.x during the index hospitalization. Serum creatinine data were unavailable, and the timing of AKI relative to LAAO could not be determined. Patients with end-stage renal disease receiving dialysis were excluded.	Conventional percutaneous LAAC; procedural contrast volume was not available in the administrative database.	AKI diagnosis coded in 1097/20,703 index hospitalizations (5.3%); onset after LAAO could not be confirmed.	CKD and multiple comorbidities were independently associated with AKI. AKI was associated with markedly increased in-hospital mortality (aOR 16.01; 95% CI 8.48–30.21) and 180-day all-cause readmission (aOR 1.43; 95% CI 1.09–1.88).	AKI identified during the index hospitalization; readmission outcomes assessed at 30, 90, and 180 days.
Zhang et al., 2022 [15]	512	AKI defined according to KDIGO serum creatinine criteria: an increase in SCr ≥0.3 mg/dL within 48 h or to ≥1.5 times baseline within 7 days. Assessment used the peak SCr value within 7 days after LAAO.	Conventional contrast-guided LAAC; mean contrast volume 146 ± 22 mL overall, 154 ± 23 mL in the AKI group versus 145 ± 22 mL in the non-AKI group (*p* = 0.051). The CV/GFR ratio was also numerically higher in the AKI group (2.3 [1.8–2.7] vs. 1.9 [1.6–2.3]; *p* = 0.055).	AKI in 27/512 patients (5.3%).	Independent predictors of AKI were lower BMI (OR 0.889; 95% CI 0.802–0.986; *p* = 0.026), hypertension (OR 5.577; 95% CI 1.267–24.558; *p* = 0.023), and CKD stages 4–5 (OR 6.729; 95% CI 1.566–28.923; *p* = 0.010). Contrast volume was not established as an independent predictor. AKI was associated with increased MACE and mortality.	AKI assessed within 7 days after LAAO; median clinical follow-up 15 months (IQR 4–26), with outcomes reported up to 3 years.
B. Studies evaluating contrast-sparing or zero-contrast LAAO						
Wisst et al., 2018 [24]	46 (21 no-contrast vs. 25 conventional)	Renal function was not systematically assessed as a study endpoint, and no study-specific AKI definition or postprocedural creatinine-monitoring protocol was reported.	Amulet LAAC without angiographic contrast versus conventional contrast-guided implantation; 0 mL contrast in the no-contrast group.	AKI and postprocedural renal-function changes were not specifically reported.	Procedural success and complication rates were comparable between groups. The study demonstrated feasibility of contrast-free LAAC but was not designed to establish renal protection.	Clinical and echocardiographic follow-up at approximately 3 months; renal follow-up was not specifically defined.
Sedaghat et al., 2019 [16]	40 (20 vs. 20)	Baseline GFR: 41 ± 21 versus 65 ± 24 mL/min; SCr: 2.5 ± 2.0 versus 1.2 ± 0.5 mg/dL in the contrast-free and conventional groups, respectively. Four patients in the contrast-free group were receiving chronic hemodialysis. No study-specific AKI definition or postprocedural creatinine assessment schedule was reported.	Contrast-free Amulet LAAO versus conventional implantation; procedural contrast volume: 0 versus 118 ± 31 mL.	AKI and postprocedural renal function changes were not specifically reported	Procedural success was 100% in both groups; contrast-free LAAO was associated with lower radiation exposure and shorter fluoroscopy time	Periprocedural assessment and clinical/echocardiographic follow-up at approximately 3 months; timing of postprocedural renal assessment not reported.
Saad Shaukat et al., 2020 [25]	12	Contrast avoidance was prompted by CKD stage IV in 5 patients, rapidly progressive CKD stage III in 1 patient, and contrast allergy in 6 patients. A study-specific AKI definition and systematic postprocedural renal-function assessment were not reported.	TEE- and fluoroscopy-guided WATCHMAN implantation without iodinated contrast; contrast volume 0 mL.	AKI and postprocedural renal-function changes were not specifically reported.	WATCHMAN implantation without contrast was successful in all 12 patients. No immediate or delayed procedural complications were reported through 45 days. The study demonstrated procedural feasibility in patients with advanced CKD or contrast allergy but did not establish renal protection.	Immediate postprocedural assessment and 45-day TEE follow-up; renal follow-up was not specifically defined.
Chu et al., 2020 [26]	14 (7 zero-fluoroscopy/zero-contrast vs. 7 propensity-matched conventional)	Baseline renal status and a study-specific AKI definition were not reported as study endpoints; no systematic postprocedural renal-function assessment was described.	ICE- and electroanatomic mapping-guided LAmbre LAAC without fluoroscopy or iodinated contrast versus conventional LAAC; contrast consumption was 0 mL in the zero-fluoroscopy group.	AKI and postprocedural renal-function changes were not separately reported.	Device implantation and acute complete LAA sealing were successful in all 7 zero-fluoroscopy patients, with no procedure-related complications. Procedural duration and follow-up peridevice leak were comparable with the propensity-matched conventional group. The study supports procedural feasibility but does not provide evidence of renal protection.	45-day follow-up reported; renal follow-up was not specifically defined.
Flautt et al., 2022 [27]	53	Renal dysfunction was recognized as a relevant clinical context for contrast avoidance, but baseline renal-function data, a study-specific AKI definition, and a systematic postprocedural creatinine-monitoring protocol were not reported.	ICE-guided LAAC without iodinated contrast in all 53 patients; 41 procedures were planned without fluoroscopy, with no bailout fluoroscopy required.	AKI and postprocedural renal-function changes were not separately reported.	LAAC was successful in all patients without procedural complications or acute peridevice leak. The study demonstrated that ICE-guided contrast-free LAAC was feasible, including a fluoroscopy-free approach in selected patients, but did not provide evidence of renal protection.	Post-implant imaging was available in 51/53 patients; the timing of renal follow-up was not specifically defined.
Zou et al., 2023 [28]	126 (32 TEE-only vs. 94 fluoroscopy-guided; propensity-matched 25 vs. 25)	Renal insufficiency was defined as eGFR <60 mL/min. The TEE-only group had lower baseline eGFR than the fluoroscopy-guided group (52.7 ± 8.8 vs. 71.8 ± 15.9 mL/min), and 78.1% versus 23.4% had eGFR <60 mL/min. A study-specific AKI definition and systematic postprocedural creatinine-monitoring protocol were not reported.	TEE-only, non-fluoroscopic LAAC in patients at high risk from contrast exposure versus conventional fluoroscopy-guided LAAC.	AKI and postprocedural renal-function changes were not separately reported.	LAAC was successfully performed in all patients. After propensity matching, procedural success and complication rates were comparable between the TEE-only and fluoroscopy-guided groups. The study supports procedural feasibility in patients at increased contrast-related risk but does not establish renal protection.	Mean follow-up 22.2 ± 4.5 months; renal follow-up was not specifically defined.
Chakravarty et al., 2023 [29]	110	Baseline SCr was 1.6 ± 1.6 mg/dL; CKD stage ≥3 was present in 58% of patients. A standardized AKI definition and postprocedural creatinine measurement schedule were not reported.	TEE-guided zero-contrast WATCHMAN FLX LAAO	Transient AKI in 6/110 patients (5.5%); no patient required hemodialysis	AKI occurred despite complete avoidance of iodinated contrast; the uncontrolled design did not permit estimation of a preventive effect	Procedural safety assessed through 7 days or hospital discharge, whichever was later; device imaging at 45 days. The AKI assessment window was not specified.
Blusztein et al., 2023 [17]	31	Renal impairment or risk considered; AKI not separately reported.	Zero-contrast LAAO using hybrid echocardiography–fluoroscopy guidance	AKI not separately reported	Procedural success was 100%; no iodinated contrast was used and no device-related complications were reported at 45 days	45 days
Fukushima et al., 2024 [18]	15	CKD stages 3b–5	Attempted zero-contrast LAAC; complete contrast avoidance in 13/15 patients; 2 patients required 6 and 15 mL of contrast	No acute kidney disease reported during follow-up	Procedural success was 100%, with no periprocedural complications; the reported renal outcome did not establish comparative protection against AKI	45 days
Okada et al., 2024 [23]	10	CKD; baseline eGFR 28 ± 12 mL/min/1.73 m^2^; preprocedural and postprocedural eGFR assessed	Contrast-free WATCHMAN FLX LAAC	No significant change in postprocedural eGFR; no acute kidney disease reported during hospitalization	Contrast-free implantation was successful in all patients, with no in-hospital adverse events; stable eGFR was not treated as equivalent to standardized AKI ascertainment	In-hospital; imaging follow-up within 3 months
Vij et al., 2024 [19]	118	Baseline GFR was 50.2 ± 22.3 mL/min and SCr was 1.58 ± 1.05 mg/dL; five patients were receiving chronic hemodialysis. Periprocedural complications were assessed with reference to VARC-2 and Munich consensus definitions, but AKI-specific operational criteria and the creatinine measurement schedule were not separately described.	Contrast-free LAMBRE LAAO	No AKI reported	Device success was 97.5%; the uncontrolled design did not establish a reduction in AKI relative to contrast-guided LAAO	Periprocedural/short-term
Ateş et al., 2026 [20]	324	AKI assessed according to KDIGO criteria during the index hospitalization; SCr reported at baseline and 24–48 h after LAAO. CKD was present in 83/324 patients (25.6%), including 19 receiving maintenance hemodialysis. The handling of maintenance hemodialysis patients in the AKI analysis was not specified.	Zero-contrast LAAC	No AKI during the index hospitalization; renal function remained stable in the CKD subgroup	Procedural success was 99.7%; the absence of a contemporaneous contrast-guided comparator limited conclusions regarding AKI prevention	SCr assessed at baseline and 24–48 h after LAAO; AKI recorded during the index hospitalization.
Cieślik et al., 2026 [30]	159 (84 vs. 75)	Renal function was relevant to the contrast-avoidance strategy; AKI not separately reported as an endpoint	Zero-contrast vs. contrast-guided LAAO	AKI not separately reported	Similar procedural safety and efficacy outcomes were reported between groups; comparative renal protection was not established	Procedural/follow-up outcomes
Demian et al., 2026 [21]	621 (326 vs. 295)	Renal outcomes not reported as a separate AKI endpoint	Zero-contrast vs. contrast-guided WATCHMAN FLX LAAO	AKI not separately reported	Similar procedural success and adverse-event rates were reported between strategies; comparative renal protection was not established	45 days and 1 year

Note: Studies are grouped according to their primary focus. Renal outcomes are presented using the terminology reported by the original investigators. Absence of a separately reported AKI endpoint was not interpreted as absence of AKI events. Changes in eGFR and reports of acute kidney disease were not treated as interchangeable with standardized AKI ascertainment. Renal outcomes from uncontrolled cohorts do not establish a preventive effect of contrast avoidance. Abbreviations: AKI, acute kidney injury; BMI, body mass index; CKD, chronic kidney disease; eGFR, estimated glomerular filtration rate; KDIGO, Kidney Disease: Improving Global Outcomes; LAAC, left atrial appendage closure; AKIN, Acute Kidney Injury Network; LAAO, left atrial appendage occlusion; MACE, major adverse cardiovascular events; OR, odds ratio; SCr, serum creatinine; TEE, transesophageal echocardiography.

## Data Availability

No new data were created or analyzed in this study. Data sharing is not applicable to this article.

## Supplementary Materials

The following supporting information can be downloaded at: https://www.mdpi.com/article/10.3390/jcm15207919/s1, Table S1. Database-specific search strategies used in the updated systematic literature search; Table S2A. Item-level risk-of-bias assessment of cohort and comparative observational studies using the revised JBI Critical Appraisal Tool for Cohort Studies; Table S2B. Item-level methodological appraisal of uncontrolled case series using the JBI Critical Appraisal Checklist for Case Series.

## Abbreviations

The following abbreviations are used in this manuscript:

AF	Atrial fibrillation
AKI	Acute kidney injury
CA-AKI	Contrast-associated acute kidney injury
CI-AKI	Contrast-induced acute kidney injury
CKD	Chronic kidney disease
eGFR	Estimated glomerular filtration rate
JBI	Joanna Briggs Institute
KDIGO	Kidney Disease: Improving Global Outcomes
LAA	Left atrial appendage
LAAO	Left atrial appendage occlusion
MACE	Major adverse cardiovascular events
PRISMA	Preferred Reporting Items for Systematic Reviews and Meta-Analyses
TEE	Transesophageal echocardiography
