# Supplementary material for: Acute Kidney Injury After Percutaneous Left Atrial Appendage Occlusion: Incidence, Risk Factors, Clinical Impact, and Contrast-Sparing Strategies—A Systematic Review
Source: J Clin Med. 2026 Oct 14;15(20):7919. doi: 10.3390/jcm15207919 (PMC13706490; doi:10.3390/jcm15207919)
Supplement: Supplementary file 1 [file jcm-15-07919-s001.zip › jcm-4595121-supplementary.pdf]

Supplementary Table S1. Database-specific search strategies used in the updated systematic literature search

Database	Search strategy	Date searched	Records
**PubMed**	("left atrial appendage occlusion" OR "left atrial appendage closure" OR "percutaneous left atrial appendage occlusion" OR "percutaneous left atrial appendage closure" OR "left atrial appendage occluder" OR LAAO OR LAAC OR Watchman OR "Watchman FLX" OR Amulet OR "Amplatzer Amulet" OR "Amplatzer Cardiac Plug" OR WaveCrest OR LAmbre OR Ultraseal) AND ("acute kidney injury" OR "acute renal injury" OR "acute kidney failure" OR "acute renal failure" OR AKI OR "chronic kidney disease" OR CKD OR "kidney disease" OR "renal disease" OR "renal insufficiency" OR "renal impairment" OR "kidney impairment" OR "renal dysfunction" OR "kidney dysfunction" OR "renal failure" OR ESRD OR dialysis OR hemodialysis OR haemodialysis OR "renal replacement therapy" OR "contrast-associated acute kidney injury" OR "contrast induced acute kidney injury" OR "contrast-induced acute kidney injury" OR "contrast-associated nephropathy" OR "contrast induced nephropathy" OR "contrast-induced nephropathy" OR "contrast nephropathy" OR nephrotoxicity OR "zero contrast" OR "zero-contrast" OR "contrast free" OR "contrast-free" OR "low contrast" OR "low-contrast" OR "contrast sparing" OR "contrast-sparing" OR "contrast minimization" OR "contrast minimisation")	6 October 2026	**247**
**Web of Science Core Collection**	**Topic search:** ("left atrial appendage occlusion" OR "left atrial appendage closure" OR "percutaneous left atrial appendage occlusion" OR "percutaneous left atrial appendage closure" OR "left atrial appendage occluder" OR LAAO OR LAAC OR Watchman OR "Watchman FLX" OR Amulet OR "Amplatzer Amulet" OR "Amplatzer Cardiac Plug" OR WaveCrest OR LAmbre OR Ultraseal) AND ("acute kidney injury" OR "acute renal injury" OR "acute kidney failure" OR "acute renal failure" OR AKI OR "chronic kidney disease" OR CKD OR "kidney disease" OR "renal disease" OR "renal insufficiency" OR "renal impairment" OR "kidney impairment" OR "renal dysfunction" OR "kidney dysfunction" OR "renal failure" OR ESRD OR dialysis OR hemodialysis OR haemodialysis OR "renal replacement therapy" OR "contrast-associated acute kidney injury" OR "contrast induced acute kidney injury" OR "contrast-induced acute kidney injury" OR "contrast-associated nephropathy" OR "contrast induced nephropathy" OR "contrast-induced nephropathy" OR "contrast nephropathy" OR nephrotoxicity OR "zero contrast" OR "zero-contrast" OR "contrast free" OR "contrast-free" OR "low contrast" OR "low-contrast" OR "contrast sparing" OR "contrast-sparing" OR "contrast minimization" OR "contrast minimisation")	6 October 2026	**312**
**Scopus**	TITLE-ABS-KEY(("left atrial appendage occlusion" OR "left atrial appendage closure" OR LAAO OR LAAC) AND ("acute kidney injury" OR AKI OR "chronic kidney disease" OR CKD OR "renal dysfunction" OR "renal impairment" OR dialysis OR "zero contrast" OR "zero-contrast" OR "contrast-free"))	6 October 2026	**269**
**Cochrane CENTRAL**	("left atrial appendage occlusion" OR "left atrial appendage closure" OR LAAO OR LAAC OR Watchman OR Amulet OR LAmbre) AND ("acute kidney injury" OR AKI OR "chronic kidney disease" OR CKD OR "renal dysfunction" OR "renal impairment" OR "renal insufficiency" OR ESRD OR dialysis OR "zero contrast" OR "zero-contrast" OR "contrast-free" OR "contrast sparing" OR "contrast-sparing")	6 October 2026	**21**

Note: Searches were performed from database inception through 6 October 2026. No language restrictions or study-design filters were applied. Search syntax was adapted to the functionality of each database. Reference lists and citation networks of eligible studies and relevant reviews were additionally examined, identifying one additional eligible report.

Supplementary Table S2A. Item-level risk-of-bias assessment of cohort and comparative observational studies using the revised JBI Critical Appraisal Tool for Cohort Studies

Study	Q1	Q2	Q3	Q4	Q5	Q6	Q7	Q8	Q9	Q10	Q11
Nombela-Franco et al., 2018	N	Y	Y	Y	Y	Y	Y	Y	N	N	Y
Wisst et al., 2018	N	Y	Y	N	N	Y	Y	Y	N	N	Y
Sedaghat et al., 2019	N	Y	Y	Y	Y	Y	Y	Y	N	N	Y
Sedaghat et al., 2020	N	Y	Y	Y	Y	Y	Y	Y	N	N	Y
Chu et al., 2020	Y	Y	Y	Y	Y	Y	Y	Y	Y	NA	Y
Nazir et al., 2021	N	Y	U	Y	Y	U	U	Y	U	U	Y
Zhang et al., 2022	N	Y	Y	Y	Y	Y	Y	Y	U	N	Y
Zou et al., 2023	Y	Y	Y	Y	Y	Y	Y	Y	Y	NA	Y
Cieślik et al., 2026	U	Y	Y	U	U	Y	Y	Y	U	U	Y
Demian et al., 2026	N	Y	Y	Y	N	Y	Y	Y	N	N	Y

Question key:

Q1. Were the two groups similar and recruited from the same population?
Q2. Were the exposures measured similarly to assign people to exposed and unexposed groups?
Q3. Was the exposure measured in a valid and reliable way?
Q4. Were confounding factors identified?
Q5. Were strategies to deal with confounding factors stated?
Q6. Were participants free of the outcome at the start of the study or at the moment of exposure?
Q7. Were the outcomes measured in a valid and reliable way?
Q8. Was the follow-up time reported and sufficient for the outcomes to occur?
Q9. Was follow-up complete, and if not, were reasons for loss to follow-up described and explored?
Q10. Were strategies used to address incomplete follow-up?
Q11. Was appropriate statistical analysis used?

Legend: Y, Yes; N, No; U, Unclear; NA, Not applicable. Judgments were made at the item level; no aggregate numerical quality score was calculated.

Supplementary Table S2B. Item-level methodological appraisal of uncontrolled case series using the JBI Critical Appraisal Checklist for Case Series

Study	Q1	Q2	Q3	Q4	Q5	Q6	Q7	Q8	Q9	Q10
Saad Shaukat et al., 2020	Y	Y	Y	Y	N	Y	Y	Y	Y	Y
Flautt et al., 2022	U	Y	Y	U	U	U	Y	Y	Y	Y
Chakravarty et al., 2023	Y	Y	Y	Y	Y	Y	Y	U	Y	Y
Blusztein et al., 2023	U	Y	Y	U	U	Y	Y	Y	Y	Y
Fukushima et al., 2024	Y	Y	Y	U	U	Y	Y	Y	Y	Y
Okada et al., 2024	U	Y	Y	U	U	Y	Y	Y	Y	Y
Vij et al., 2024	Y	Y	Y	U	U	Y	Y	Y	Y	Y
Ateş et al., 2026	Y	Y	Y	Y	Y	Y	Y	Y	Y	Y

Question key:

Q1. Were there clear criteria for inclusion in the case series?
Q2. Was the condition measured in a standard and reliable way for all participants?
Q3. Were valid methods used to identify the condition in all participants?
Q4. Did the case series include participants consecutively?
Q5. Did the case series include participants completely?
Q6. Were participant demographics clearly reported?
Q7. Was relevant clinical information clearly reported?
Q8. Were outcomes or follow-up results clearly reported?
Q9. Was the demographic information of the presenting site(s)/clinic(s) clearly reported?
Q10. Was the statistical analysis appropriate?

Legend: Y, Yes; N, No; U, Unclear; NA, Not applicable. Judgments were made at the item level; no aggregate numerical quality score was calculated.
